# Combination of Paclitaxel and PXR Antagonist SPA70 Reverses Paclitaxel-Resistant Non-Small Cell Lung Cancer

**DOI:** 10.3390/cells11193094

**Published:** 2022-10-01

**Authors:** Xiaxia Niu, Ting Wu, Qishuang Yin, Xinsheng Gu, Gege Li, Changlong Zhou, Mei Ma, Li Su, Shu Tang, Yanan Tian, Ming Yang, Hongmei Cui

**Affiliations:** 1 Institute of Toxicology, School of Public Health, Lanzhou University, Lanzhou 730000, China; 2 State Key Laboratory of Applied Organic Chemistry, College of Chemistry and Chemical Engineering, Lanzhou University, Lanzhou 730000, China; 3 College of Basic Medical Sciences, Hubei University of Medicine, Shiyan 442000, China; 4 Department of Veterinary Physiology and Pharmacology, Texas A&M University, College Station, TX 77843, USA

**Keywords:** PTX resistance, PXR, Tip60, SPA70, acetylation of α-tubulin, mitosis defect

## Abstract

Paclitaxel (PTX) is one of the most efficient drugs for late-stage non-small cell lung cancer (NSCLC) patients. However, most patients gradually develop resistance to PTX with long-term treatments. The identification of new strategies to reverse PTX resistance in NSCLC is crucially important for the treatment. PTX is an agonist for the pregnane X receptor (PXR) which regulates PTX metabolism. Antagonizing PXR, therefore, may render the NSCLC more sensitive to the PTX treatment. In this study, we investigated the PXR antagonist SPA70 and its role in PTX treatment of NSCLC. In vitro, SPA70 and PTX synergistically inhibited cell growth, migration and invasion in both paclitaxel-sensitive and paclitaxel-resistant A549 and H460 lung cancer cells. Mechanistically, we found PTX and SPA70 cotreatment disassociated PXR from ABCB1 (MDR1, P-gp) promoter, thus inhibiting P-gp expression. Furthermore, the combination regimen synergistically enhanced the interaction between PXR and Tip60, which abrogated Tip60-mediated α-tubulin acetylation, leading to mitosis defect, S-phase arrest and necroptosis/apoptosis. Combination of PXT and SPA70 dramatically inhibited tumor growth in a paclitaxel-resistant A549/TR xenograft tumor model. Taken together, we showed that SPA70 reduced the paclitaxel resistance of NSCLC. The combination regimen of PTX and SPA70 could be potential novel candidates for the treatment of taxane-resistant lung cancer.

## 1. Introduction

Lung cancer is the most lethal cancer in the world and the leading cause of cancer death [1]. Over 80~85% of lung cancer types are non-small cell lung cancers (NSCLC), and the 5-year survival rate of NSCLC in stage IV is less than 18% [2]. Some patients with later stages of NSCLC respond to and benefit from immunotherapy using immune checkpoint inhibitors (ICIs) (e.g., ipilimumab, atezolizumab, nivolumab) to disrupt T-lymphocyte-associated protein 4 (CTLA-4) or programmed cell death protein 1 (PD-1)/programmed cell death protein 1 ligand (PD-L1) pathways, thus reactivating T-cell response to kill tumor cells [3]. However, chemotherapy and targeted therapy are still the main strategies for the majority of lung cancer patients [3,4].

As one of the most efficient clinical chemotherapeutic drugs for lung cancer, paclitaxel (PTX) arrested the tumor cell cycle in the G2/M phase, leading to cancer cell death through stabilization of microtubules [5,6,7]. However, long-term treatment with PTX results in drug resistance though overexpressed P-glycoprotein (P-gp, encoded by gene ABCB1/MDR1), altered tubulin acetylation, and other resistance mechanisms [8]. A regimen targeting overexpressed P-gp and β-tubulin-III (TUBB3) in resistant sublines [9], interfering with microtubule dynamics and spindle checkpoint [10], or inhibiting HDAC1 activity [11], has been proven to exert reversal effects on PTX resistance. It has been reported that the acetylation status of α-tubulin manipulates the microtubule dynamics, and MYST histone acetylate transferase (HAT) Tip60 affects α-tubulin acetylation levels of microtubules [12], therefore, Tip60 and its regulatory mechanisms may be involved in PTX resistance.

Over 50% of xenobiotics, including a wide range of therapeutic drugs, are metabolized through the pregnane X receptor (PXR, NR1I2)-regulated metabolic detoxification system. PTX has been shown to be an agonist of PXR, which activates CYP3A4 and MDR1 gene expression [13,14,15]. The PXR is a ligand-dependent orphan nuclear receptor that is important for drug metabolism, and as such plays a major role in the process of therapeutic resistance during cancer treatment [16,17,18,19]. The PXR is enriched in liver and colon tissue [20], however, NSCLC cells such as A549 and H460 cells also contain PXR, suggesting that PXR plays a role in regulating drug resistance in lung cancer therapeutic resistance [14,15,21,22]. Antagonizing PXR, therefore, may render the lung cancer cells sensitive to the PTX treatment.

**Figure 1:**
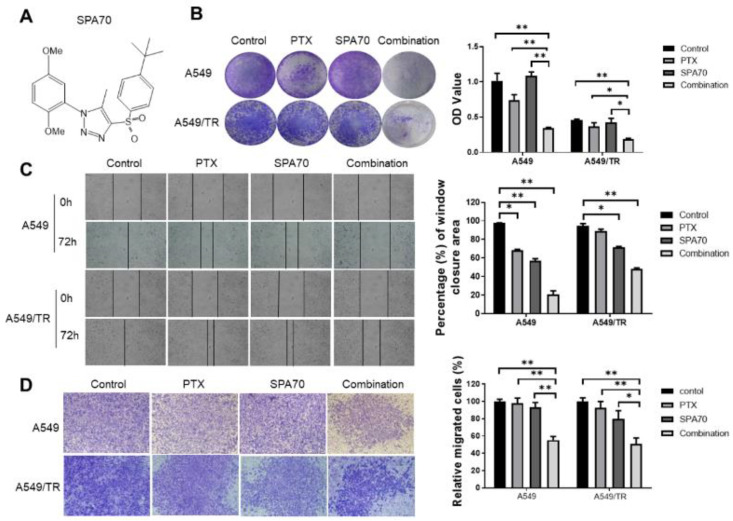
The effect of PTX, SPA70 and combination treatment on the cell colony formation and migration, invasion effects of PTX, SPA70 and combination on A549, A549/TR cells. (**A**) Chemical structure of SPA70. (**B**) Representative images from the colony formation assay, and the quantification of the results from A549, A549/TR. The experiment was conducted using 1 nM PTX, 5 μM SPA70 in 12-well plates with triplicates. Colonies were lysed in buffer with SDS and read the absorbance at 490 nm. Quantification was applied to show relative cell viability. One-way ANOVA test was performed for statistical significance. *, *p* < 0.05; **, *p* < 0.01, compared with indicated groups. (**C**) Wound healing assay was carried out in 12-well plates with triplicates and the photo was taken after drugs were added for 72 h. The drug concentration was 2 nM PTX and/or 10 μM SPA70. The migrated cells that crossed the scratch line were quantified and displayed as percentage of total wound closure area. One-way ANOVA test was performed for statistical significance. *, *p* < 0.05; **, *p* < 0.01, compared with indicated groups. The photographs were taken by microscopy (200×). (**D**) 2 nM PTX and/or 10 μM SPA70 were treated for 48 h and pictures were taken from the opposite side of the Matrigel-coated surface, which indicates the invaded cells to the lower chamber. The photographs were taken by microscopy (200×). Quantification was applied to show cell invasion. One-way ANOVA test was performed for statistical significance. *, *p* < 0.05; **, *p* < 0.01, compared with indicated groups.

SPA70 (1-(2,5-Dimethoxyphenyl)-4-[[4-(1,1-dimethylethyl)phenyl]sulfonyl]-5-methyl-1H-12,3-triazole,LC-1) (Figure 1A) has been shown to be a very potent and specific PXR antagonist [23]. It dramatically inhibits rifampicin (RIF)-induced Cyp3A expression, while strengthens the RIF-induced protein expression of PXR [23]. Additionally, SPA70 augments the association of PXR with co-repressors (NCoR and SMRT), while it has no effects on the association of co-activators (SRC-1 or TIF2) with PXR [23]. PXR protein contains 434 amino acids, including N-terminal ligand-independent activation function 1 (activation function 1, AF1), highly conserved DNA-binding domain (DBD), hinge region, C-terminal ligand binding domain (LBD) and activation function 2 domain (AF-2) [24,25]. Ligand-bound PXR forms heterodimers with the retinoid x receptor (RXR), then is translocated from the cytoplasm to the nucleus to initiate the downstream gene’s transcription [26]. The drug-resistant gene MDR1 has a direct repeats (DR)-4 responsive element in the promoter region and can be recognized and transcriptionally regulated by PXR [27,28]. Overexpression of PXR and MDR1 was observed in PTX-resistant NSCLC sublines and highly correlated with PTX resistance [29,30]. Therefore, PXR antagonists bearing the potential to inhibit PXR-regulated P-gp expression offer a promising strategy to reverse PTX resistance [31,32,33]. Recent studies showed that PXR played a role in chromosome segregation during mitosis [34]. Structurally, there is a nuclear localization sequence (NLS) and two zinc-finger structures of C4 type residing in the DBD region [34,35]. The region of PXR (R66-76R) governs its nuclear translocation and regulates PXR interaction with the mitotic chromatin [34]. Additionally, the function of PXR is finely tuned by certain transcriptional co-regulators. The agonist binding of PXR causes conformational changes of PXR, leading to disassociation of co-repressors (HDAC/SMRT/NCoR) and recruitment of co-activators, such as steroid receptor co-activator 1 (SRC-1) and RXRα [36,37,38,39]. Co-activators display HAT activity to loosen the highly condensed chromosome structure, thus facilitating PXR transcriptional activation [39]. A recent study showed that PXR augmented the HAT activity of Tip60 and promoted colon cancer cell adhesion and migration [40]. Therefore, fine tuning the PXR-Tip60 activity by PXR antagonist may overcome PTX resistance.

In this study, we examined the in vivo and in vitro effect of the combination regimen of PTX and the specific PXR antagonist SPA70 on PTX-sensitive and PTX-resistant NSCLC subline A549 and H460. Furthermore, in order to investigate whether SPA70 derivatives also bear clinical potential to overcome PTX resistance, we developed two analogues based on the scaffold of SPA70 and evaluated their efficiency. Understanding how the PXR antagonist reverses multidrug resistance and ascertaining how PXR regulates cellular mitosis will help us reveal the role of PXR in regulating drug resistance and develop an effective strategy for the treatment of taxane-resistant lung cancer.

## 2. Materials and Methods

### 2.1. Cell Culture and Reagents

A549 and H460 cells (NSCLC) from American Type Culture Collection (ATCC, Manassas, VA, USA) were cultured in RPMI 1640 medium (BOSTER, China) containing 10% fetal bovine serum (Biological Industries, Shanghai, China) and 1% penicillin/streptomycin solution (Meilunbio, Dalian, China). Cells were tested to be mycoplasma-free by using a Mycoplasma Stain Assay Kit (Beyotime, C0296) (Contains Hoechst) according to the manufacturer’s instructions.

PTX-resistant A549/TR cells and H460/TR cells were obtained by selection with PTX. We started with 10 nM PTX. Briefly, A549 and H460 cells were cultured in RPMI complete medium with 10 nM PTX and then in PTX-free medium after dead cells were removed. After the cells recovered, the cells were cultured in medium containing an increased concentration of PTX, and then in PTX-free medium after dead cells were removed for recovery. The process was repeated for three months. Stable A549/TR (IC_50_ = 0.50 μM) and H460/TR cells (IC_50_ = 0.80 μM) were generated. Resistant index = IC_50_ of the PTX-resistant cell/IC_50_ of the parental cell.

To generate PXR knockdown cells, PXR KO plasmid (sc-400824-KO-2) and PXR HDR plasmid (sc-400824-HDR-2) were introduced into A549/TR cells by transfection. A total of 0.5 μg/uL puromycin was used for selection for 2 days. Next, the PXR knockdown pool clones were collected and confirmed using Western blotting.

Flag-Tip60 and GST-Tip60 was kindly provided by Dr. Yi Tang (Albany Medical College). Transfections were carried out according to the manufacturer’s instructions.

PTX (APExBIO, A4393), SPA70 (sigma, SML2662) (Figure 1A) were dissolved in DMSO (ATCC) at a stock solution of 20 mM and stored in −20 °C in a freezer.

### 2.2. Cell Proliferation and IC_50_ Measurement

Around 5000 cells in 50 μL cell culture medium per well were seeded in 96-well plates. A stock solution of 20 mM PTX or SPA70 was diluted to 0.002~60 μM. On the next day, 50 μL cell culture medium containing the indicated compound at different concentrations was added into the well to make the final drug concentration ranging from 0.001~30 μM. After 72 h, 10 μL CCK-8 solution (APExBIO, K1080) was added for 1~4 h and cell viability was measured with absorbance at 490 nm. The OD value was input to the GraphPad Prism software 8.0.2 (San Diego, CA, USA) and the half maximal inhibitory concentration (IC_50_) values were calculated.

### 2.3. Colony Formation Assay

Around 1000 cells were seeded with triplicates in 12-well plates and were treated with different compounds. After 8~10 d, surviving colonies were fixed with 10% formalin and then stained with 0.1% of crystal violet. After washing, stained colonies were dissolved with buffer containing SDS, and measured by a microplate reader at 490 nm absorbance.

### 2.4. Wound Healing Assay and Invasion Assay

Wound healing assay was used to determine the migration potential of A549 or H460 cells. Around 10,000 A549 or H460 and A549/TR or H460/TR cells were firstly seeded into 12-well plates with triplicates until attached. Next, a 200 μL pipet tip was used to make a straight line through the attached cells in order to create uniform identical scratches. After cellular debris was removed, the fresh new medium containing PTX (2 nM), SPA70 (10 μM), PTX (2 nM) + SPA70 (10 μM) was added. At 72 h after drug treatment, the photographs were taken. In the cell invasion assay, cells in a FBS-free medium were added into the top chamber which contained Matrigel (24-well Matrigel Invasion Chambers, Corning, Biocoat), and treated with different compounds. In the meantime, 10% FBS as an attractant was added in the lower chamber. After 48 h, the invaded cells on the bottom of the upper chamber were fixed and stained with crystal violet, and captured by a microscope.

### 2.5. Intracellular Rh-123 Uptake

In order to test P = −gp transporter activity, we measured Rhodamine-123 (Rh-123) accumulation in A549/TR cells by flow cytometric analysis (FACS). Cells were seeded in 6-well plates, and treated with compounds, together with 10 μM Rh-123, for 48 h. After washing with PBS, cells were re-suspended for FACS analysis. We also used a fluorescence microscope to confirm Rh-123 uptake in the cells that attached on glass coverslip.

### 2.6. ChIP Assay

A ChIP assay kit (Beyotime, P2078) was used to perform ChIP assay. Briefly, A549/TR cells were treated with the indicated regimen for 48 h, then were cross-linked with formaldehyde and processed following the manufacturer’s recommendation. The precipitated DNA product was analyzed by PCR in triplicates with three independent experiments. The MDR1 promoter fragment (−7975 to −7013) containing the cluster of PXR response elements [28] was amplified by PCR with primers: forward 5′-TCATGGTCTGCTAGCAGTGT-3′ and reverse 5′-ACCAAACCCTTTGCCCTAAGA-3′.

### 2.7. Flow Cytometric Measurement of Cell Cycle and Apoptosis

The cells were starved in serum-free medium for 24 h, then were treated with paclitaxel (2 nM) and/or SPA70 (10 μM). At 48 h after treatment, cells were pre-fixed in ice-cold ethanol (70%) overnight, then washed with PBS on the second day and incubated with a staining buffer containing 50 μg/mL propidium iodide (PI), and 10 μg/mL RNase for 1 h at dark, and analyzed by flow cytometry. For apoptosis assays, the cells were incubated with annexin V-FITC reagents in the kit (Elabscience, annexin V-FITC/PI fluorescent double staining apoptosis detection kit). The data was acquired by a Bio-Rad ZE5 flow cytometer and was analyzed by ModFit LT 2.0 and FlowJo v10.4 software.

### 2.8. Immunofluorescence Staining

A549/TR cells were treated with the indicated compounds for 48 h, and then were fixed with fresh-made 4% paraformaldehyde (PFA) and permeabilized with 0.2% Triton X-100 in PBS solution. α-tubulin antibody (Thermo Scientific, Waltham, MA, USA, 1:400) was used to stain microtubules, and then labeled with Alexa Fluor 647 goat anti-mouse IgG (Santa cruz) at dark for 1 h. The coverslips were washed with PBS and mounted in a glass slide with anti-fade media containing DAPI (Vectorlabs, H-1200). The photographs were taken with a fluorescent microscope (Olympus BX-53).

### 2.9. Real-Time Quantitative PCR

Total RNA was extracted by TRIzol (Life Technologies Corporation) and was reverse-transcribed to complete DNA by using a reverse transcriptase kit (Takara). The cDNA as templates were used for real-time quantitative PCR. Amplifications were performed in the iQ5 Optical Module (Bio-Rad) by using SYBR Green Master Mix (Applied Biosystems). The TNFα PCR primers used were forward 5′-CCTCTCTCTAATCAGCCCTCTG-3′ and reverse 5′-GAGGACCTGGGAGTAGATGAG-3′. The MLKL PCR primers used were forward 5′-AGGAGGCTAATGGGGAGATAGA-3′, and reverse 5′-TGGCTTGCTGTTAGAAACCTG-3′. The NF-κB PCR primers used were forward 5′-GAACTCCTCCATTGTGGAACC-3′ and reverse 5′-TCGGAAGCCTCTCTGCTTAG-3′. The GAPDH was used as a housekeeping gene for normalization, and the primers were forward 5′-AACGGATTTGGTCGTATTGGG-3′, and reverse 5′-CCTGGAAGATGGTGATGGGATT-3′.

### 2.10. Western Blotting, GST-Pulldown and Co-IP

Cells were lysed in modified RIPA buffer and protein was extracted. The proteins were separated in SDS-PAGE gels and detected using corresponding antibodies. The antibodies and the dilution ratios were: PXR (Santa Cruz, Santa Cruz, CA, USA, sc-48340, 1:1000), Tip60 (Cell Signaling, Danvers, MA, USA, #12058, 1:1000), HA antibody (Santa Cruz, sc-805, 1:200), FLAG (Millipore Sigma, Burlington, MA, USA, F3165, 1:4000), β-actin (Millipore Sigma, A5441, 1:5000), p-AKT (Ser473) (Cell Signaling, #9271, 1:1000), AKT (Cell Signaling, #9272, 1:1000), cleaved PARP (Cell Signaling, #9185, 1:1000), P-gp (Beyotime, Shanghai, China, AF2245, 1:1000), acetylated lysine (Cell Signaling, #9441, 1:1000), and Acetyl-α-Tubulin (Lys40) (Cell Signaling, #3971, 1:1000). For GST-pulldown assays, IPTG-induced GST or GST fusion proteins were incubated with in vitro translated proteins (5 μL) in a buffer containing 100 mM NaCl at 4 °C overnight. On the next day, after extensive washes with a similar buffer containing 150 mM NaCl, GST agarose beads (Sigma)-bound proteins were eluted by boiling in the loading buffer and determined by Western blotting. Every Western blot was repeated three times and the protein band density was quantified by Image J to analyze statistical significance. For co-IP assay, cell lysates were incubated with the indicated antibody in RIPA buffer containing 100 mM NaCl at 4 °C overnight. Protein A/G agarose (Sigma) was then added at 4 °C for another 5 h. After extensive washes with the same RIPA buffer containing 150 mM NaCl, bounded proteins were boiled and detected by Western blotting.

### 2.11. Animal Study

All animal experiments followed the protocol that was approved by the Institutional Animal Care and Use Committee (IACUC) of Lanzhou University (China). 16 BALB/c-Nude male mice (6–8 weeks) were provided by Hunan SJA Laboratory Animal Co., Ltd. A total of 1 × 108/100 μL A549/TR cells were suspended in a solution of PBS mixed with Matrigel (BD Matrigel™ Basement Membrane Matrix, #356234) at a ratio of 2:1 right before use. A total of 100 μL of mixture was inoculated subcutaneously to the right-side dorsal flank of each mouse. Once the tumor reached around 100 mm^3^, drugs were injected into the mouse. Tumor volume was calculated as length × width 2 × 0.5. PTX and SPA70 were diluted in a vehicle solution (90% PBS, 5% PEG300 and 5% ethanol). All the compounds were applied through intraperitoneal injection, three times per week for four continuous weeks. Four groups were used in this animal study, including vehicle treatment, 5 mg/kg PTX, 30 mg/kg SPA70, and combination group (5 mg/kg PTX + 30 mg/kg SPA70). At the end of the experiments, the mice were euthanized, and tumor xenografts were isolated for pathologic analysis. One-way ANOVA was used to statistically compare tumor size and body weight for in vivo xenograft study. Tumor growth inhibition (TGI) was calculated according to the method which was previously reported [41].

### 2.12. Immunohistochemistry

The isolated tumor tissues and other major organs (heart, liver, spleen, lung, kidney) were collected and fixed in 10% formalin solution and embedded in paraffin. The antibodies were used with rabbit anti-Ki67 (#9027, Cell Signaling Technology), rabbit anti-cleaved-caspase 3 (#9664, Cell Signaling Technology), P-gp (AF2245, Beyotime Biotechnology), PXR (sc-48403, Santa Cruz Biotechnology, Inc.), Tip60 (#12058, Cell Signaling Technology). Slides were imaged with microscope and analyzed by image J.

### 2.13. Synthesis of YM-1 and YM-2

Briefly, sulfonyl chloride SI-1 (5.00 g, 16.5 mM) was added with Na_2_SO_3_ (4.17 g, 33.1 mM) and NaHCO_3_ (2.78 g, 33.1 mM) to form sodium sulfinate SI-2. SI-2 in DMF (58 mL) was added to chloroacetone (2.75 mL, 34.5 mM), stirred for 12 h at room temperature, and then saturated aqueous NaHCO_3_ (100 mL) was added. The resulting mixture was extracted with EtOAc (3 × 100 mL), and the combined organic phases were washed with brine, dried over with anhydrous MgSO_4_, filtered, and concentrated under reduced pressure. The residue was purified by flash chromatography on silica gel to create sulfonyl acetone SI-3. SI-3 (1.00 g, 3.09 mM) and MeONa (333 mg, 6.17 mM) in MeOH (10 mL) were added to a solution of azide SI-4 (663 mg, 3.70 mmol) in MeOH (2 mL) over 2 min. The reaction mixture was heated to 85 °C and stirred for 10 h. Upon completion, the reaction was quenched with saturated aqueous NaHCO_3_ (20 mL), and the resulting mixture was extracted with DCM (3 × 50 mL). The combined organic phases were washed with brine, dried over anhydrous MgSO_4_, filtered, and concentrated under reduced pressure. The residue was purified by flash chromatography to develop triazole YM-1 (223 mg, 15% yield) as a white solid. PhMgBr (31.0 µL, 2.0 M in THF, 0.06 mM) was added to the solution of triazole YM-1 (10.0 mg, 0.02 mM) and Pd (PPh_3_)_4_ (4.8 mg, 0.004 mM) in THF (0.4 mL) at 0 °C. The reaction was warmed to room temperature and stirred for 2 h before it was quenched slowly with saturated aqueous NH_4_Cl (2 mL). The resulting mixture was extracted with EtOAc (4 × 5 mL). The isolated organic phases were washed with brine, dried over anhydrous MgSO_4_, filtered, and concentrated under reduced pressure. The residue was purified by flash chromatography to synthesize triazole YM-2 (7 mg, 78% yield) as a white solid.

### 2.14. Statistical Analysis

Data were analyzed using GraphPad Prism software 8.0.2 (San Diego, CA, USA). Student’s *t*-test or one-way ANOVA were used to compare the statistical significance between indicated groups.

## 3. Results

### 3.1. SPA70 Displays Potent Cytotoxicity and Synergistic Effect with PTX in NSCLC Cells Including PTX-Resistant Cell Line

Cell viability assays were carried out to evaluate the efficacy of PTX and SPA70 on parental A549, H460 and PTX-resistant sublines A549/TR and H460/TR. As expected, PTX exhibited the highest potency in parental cells yet had no effect on PTX-resistant cell lines (A549/TR and H460/TR) (Table 1). In comparison, SPA70 displayed similar potency to induce cell death on both parental and resistant lung cancer cells, and the resistance index (RI) was small (A549/TR compared with A549, RI = 2.33; H460/TR compared with H460, RI = 1.53), indicating that SPA70 could overcome clinically relevant PTX resistance in lung cancer. We calculated the combination index (CI) to evaluate whether PTX and SPA70 have a synergistic effect. The result showed that the CI value for PTX and SPA70 was less than 1.0 in both PTX-sensitive and PTX-resistant cells (Table 2), indicating that a combination regimen with PTX and SPA70 has a strong synergistic effect in tested cell lines.

The combination of PTX and SPA70 significantly suppressed the colony formation in A549 (22.8% of control, *p* = 0.003) and H460 (44.9% of control, *p* = 0.003) cells (Figure 1B and Supplemental Figure S1A). Similarly, combination treatment retained the highest drug effects to inhibit colony formation in A549/TR (40.0% of control, *p* = 0.002) and H460/TR (43.6% of control, *p* = 0.006) cells (Figure 1B and Supplemental Figure S1A). Strikingly, after 72 h, the combination of PTX and SPA70 showed significantly higher inhibition on wound closure in A549/TR and H460/TR cells (50.8%, *p* = 0.004 and 50.6%, *p* = 0.001) compared with the control group (Figure 1C and Supplemental Figure S1B). These results suggested that combination treatment has great potential to abrogate cancer cell migration in clinically taxane-resistant relevant phenotypes. Additionally, the cell invasive capability was also determined by invasion assay. The combination regimen showed a remarkable inhibition of cell invasion in comparison with the vehicle control in tested cells (A549: *p* = 0.001; A549/TR: *p* = 0.005) (Figure 1D). These results suggested that SPA70 synergized with PTX inhibits tumor cell proliferation, migration and invasion in both PTX-sensitive and PTX-resistant lung cancer cells.

### 3.2. PTX with SPA70 Together Inhibits PXR-Mediated Transcriptional Regulation of P-gp

**Figure 2:**
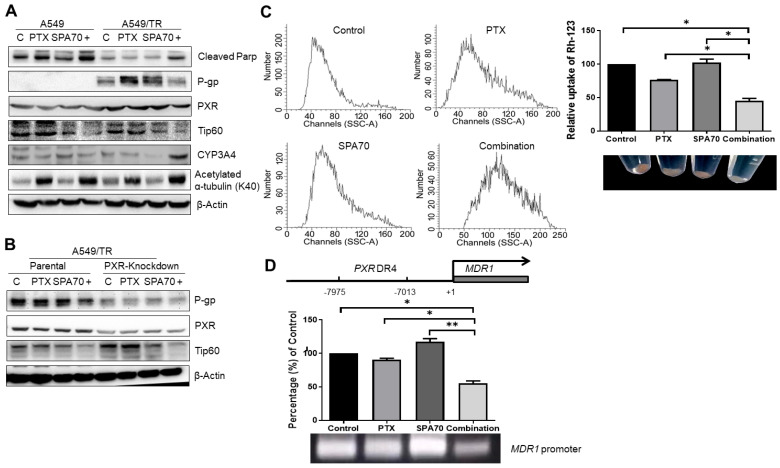
Combination regimen of PTX and SPA70 inhibits P-gp expression in PTX-resistant A549 cells. All the experiments were conducted with 2 nM PTX, 10 μM SPA70, or combined usage for 48 h (*n* = 3). (**A**) Validation of PTX-resistant A549 cells, indicated as P-gp expression, and signaling pathway analysis in both A549 and A549/TR cells upon corresponding treatment. “+” indicates combination treatment of PTX and SPA70. Protein expression was quantified by blot density as shown in Supplemental Figure S2A. (**B**) PXR knockdown decreased P-gp expression whereas it increased Tip60 protein expression. “+” indicates combination treatment of PTX and SPA70. Protein expressions was quantified by blot density as shown in Supplemental Figure S2B. (**C**) Rhodamine-123 is a P-gp substrate and indicator for *MDR1* transporter activity. FACS analysis was carried out in A549/PTX cells and lower accumulation of Rh-123 resulting from repressed P-gp was observed in the combination treatment group. Rh-123-stained cells were quantified by Modfit LT software and shown as a bar graph on the right. (**D**) ChIP assay was performed to investigate whether combination treatment disassociated *PXR* from *MDR1* promoter. *, *p* < 0.05; **, *p* < 0.01, compared with indicated groups.

**Figure 3:**
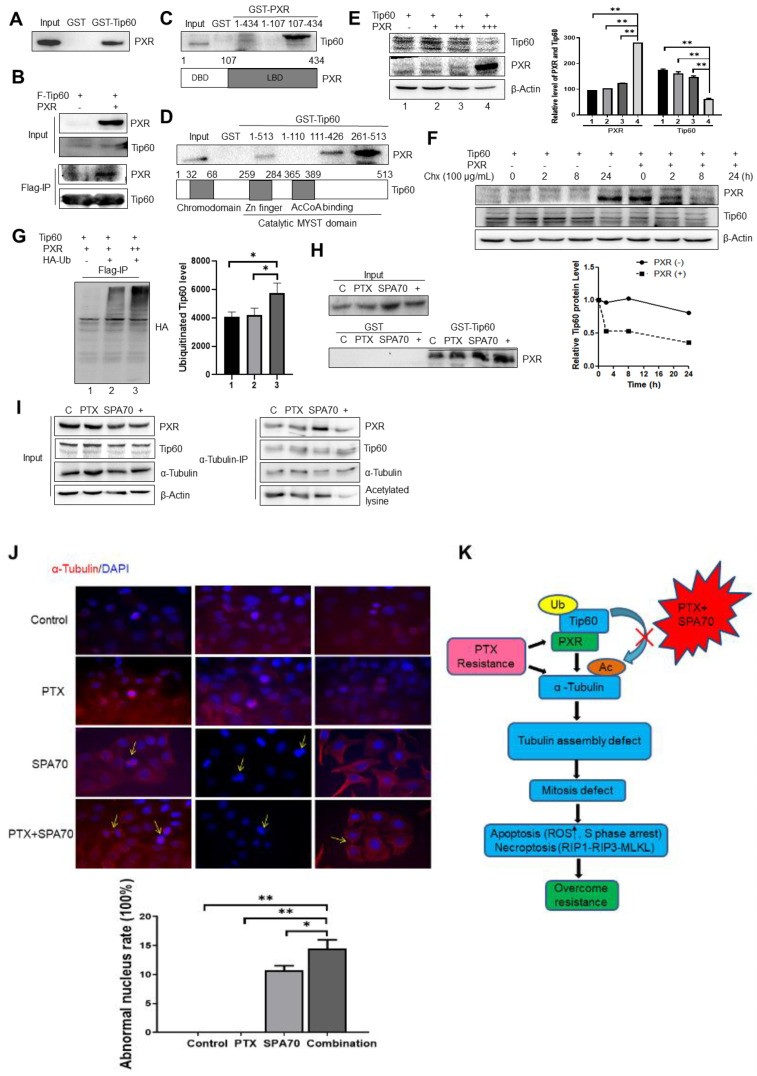
PXR interacted with Tip60 and PXR-degraded Tip60 was implicated in PTX-resistance mechanisms. (**A**) GST-Tip60 precipitated in vitro translated PXR. A total 20 μL of glutathione agarose conjugated GST-Tip60 or GST was incubated with 5 μL of in vitro translated PXR for GST-pulldown assays. (**B**) Reciprocal co-IP assay revealed that PXR interacted with Tip60. H1299 cells were co-transfected with FLAG-Tip60 (3 μg) and PXR (3 μg), and 1 μg of the FLAG antibody was used to perform the assay. (**C**) Ligand binding domain of PXR (107–434 aa) was required for Tip60 binding. PXR fragments (1 μg) were conjugated with GST and incubated with in vitro translated Tip60 for GST-pulldown assays. (**D**) Catalytic MYST domain of Tip60 binds with PXR. Tip60 truncated proteins (1 μg) conjugated with GST agarose beads and incubated with in vitro translated PXR (5 μL) for GST-pulldown assays. Bounded fragments on beads were determined by Western blotting using the PXR antibody. (**E**) PXR co-expression decreased the Tip60 expression level. FLAG-Tip60 (2 μg), and/or 0, 2, 6 μg of PXR were co-transfected to H1299 cells for 2 days, and then subjected to Western blotting as indicated. (**F**) PXR reduced Tip60 stability. Tip60 co-transfected with PXR or without PXR in H1299 cells were applied with 100 μg/mL of cycloheximide (CHX), and lysed to detect the half-life of Tip60. Relative Tip60 expression levels were quantitated in the lower plot. (**G**) PXR protein increased the Tip60 ubiquitination level. FLAG-Tip60 (2 μg), HA-ubiquitin (2 μg), and/or PXR (2, 4 μg) were transfected to H1299 cells and subjected to Flag-IP using the FLAG antibody followed by Western blotting to detect ubiquitinated proteins by the HA antibody. (**H**) Combination regimen increased binding between PXR and Tip60. Lysates prepared from A549/TR cells treated with 2 nM PTX, 10 μM SPA70, or combination regimen, were incubated with GST-Tip60 or GST for pulldown assays. “+” indicates combination treatment of PTX and SPA70. (**I**) Combination regimen-inhibited Tip60 HAT activity decreased α-tubulin acetylation. Lysates prepared from A549/TR cells treated with 2 nM PTX, 10 μM SPA70, or combination regimen, were precipitated with α-tubulin antibody, and blot with indicated antibody. “+” indicates combination treatment of PTX and SPA70. (**J**) SPA70 and combination regimen produced multipolar nucleus in PTX-resistant A549/TR cells. Immunofluorescence staining revealed that the mitosis segregation was disrupted after treatment with 10 μM SPA70, 2 nM PTX plus 10 μM SPA70 compared with control and PTX-treated cells. Chromosome segregation was visualized by immunofluorescence staining with α-tubulin (Red) and nucleus (DAPI). Multipolar nuclear and bridged α-tubulin appeared in SPA70-treated and combined treatment group. Quantification is shown in the lower panel. The photographs were taken by fluorescence microscopy (200×). (**K**) Graph illustrates the mechanisms by which PXR manipulated Tip60 degradation in PTX-resistant lung cancer cells and PXR-degraded Tip60 repressed α-tubulin acetylation after combination treatment. Consequently, decreased acetylation of α-tubulin affects tubulin dynamics and induces mitosis defect and mitosis catastrophe, leads to cell apoptosis and necroptosis, and thus sensitizes PTX-resistant lung cancer cells and overcomes drug resistance. *, *p* < 0.05; **, *p* < 0.01, compared with corresponding groups.

It is well documented that overexpression of transmembrane efflux protein P-gp leads to drug resistance [8,14]. We further determined P-gp expression in these PTX-resistant cell lines to test whether our developed A549/TR and H460-TR sublines have clinically relevant drug resistance properties. The results demonstrated that PXR and P-gp were indeed highly expressed in A549/TR and H460/TR cells (Figure 2A,B, Supplemental Figure S2A–C). SPA70 was shown to have a dramatically inhibitory effect on CYP3A mRNA and protein levels in LS180 cells [23]. Consistently, in A549/TR cells, CYP3A4 protein levels were also strongly inhibited (Figure 2A, Supplemental Figure S2A). We also noticed elevated PXR expression in resistant cell lines, and knockdown of PXR correspondingly decreased with P-gp expression (Figure 2B, Supplemental Figure S2B), which is consistent with the role of PXR in the regulation of P-gp [27]. The combined administration of PTX and SPA70 inhibited P-gp protein expression (Figure 2A, Supplemental Figure S2A). Since Rh-123 acts as a P-gp substrate and it can serve as an indicator of efflux activity of P-gp, flow cytometry analyses revealed that there was a lower accumulation of Rh-123 in the combination treatment group (Figure 2C), which seems contradictory with decreased P-gp expression. However, there was remarkable cell death induced by the combination treatment (Figure 1B), and both uptake of Rh-123 and P-gp activity might be abrogated, which explains the parallel changes of P-gp and Rh-123 accumulation in A549/TR cells. Furthermore, as shown in Figure 3C, SPA70 had no effect on Rh-123 accumulation, which excluded the possibility that SPA70 is a P-gp substrate. We next performed chromatin immunoprecipitation assay (ChIP) assays to analyze the effects of combination treatment on the regulatory effects of PXR in the MDR1 promoter; our results revealed that the combination treatment indeed disassociated PXR from the promoter region of MDR1, leading to dramatically diminished mRNA expression of MDR1 (60% of that in the control group) (Figure 2D). Western blot results revealed that the combination regimen dramatically increased the expression level of cleaved PARP in both sensitive and resistant cell lines (Figure 2A), suggesting the apoptosis of the cancer cells resulted from combination treatment.

### 3.3. PXR Degraded Tip60 and Tip60-Acetylated α-Tubulin Was Diminished by the Combination Regimen

Compared with parental cell lines, diminished HAT enzyme Tip60 expression was observed in PTX-resistant cell lines, whereas elevated Tip60 appeared when PXR was knocked down. This observation indicated that PXR-regulated Tip60 expression plays a role in PTX resistance (Figure 2A,B). Indeed, there was direct and specific interaction between PXR and Tip60 as determined by the co-immunoprecipitation assay, while agarose beads conjugated with either GST or IgG antibody failed to pull down PXR (Figure 3A,B). Domain mapping results indicated that Tip60 bound to the LBD of PXR through the Tip60 catalytic MYST domain including acetyl-CoA binding domain (Figure 3C,D). Co-expression with PXR decreased Tip60 protein expression in H1299 cells (Figure 3E). Furthermore, PXR shortened Tip60 protein half-life and destabilized Tip60 (Figure 3F). We next measured the Tip60 ubiquitination level in the presence of PXR. We found that PXR expression dramatically increased the amount of ubiquitinated Tip60 protein level (Figure 3G). Therefore, we concluded that PXR destabilized Tip60 by augmenting its ubiquitination level. In order to determine whether the combination regimen affects the binding between PXR and Tip60, we subjected cells to PTX, SPA70, and combination treatment, and incubated cell lysates with either GST or GST-Tip60 for endogenous pulldown assays [42]. The results showed that the interaction between PXR and Tip60 was enhanced by combination treatment (Figure 3H). Since Tip60 is a MYST histone acetylate transferase and it affects α-tubulin acetylation [12], we aimed to investigate whether PXR-Tip60 interaction affects α-tubulin acetylation. By using α-tubulin antibody to precipitate total acetylated lysine, we found that the acetylated α-tubulin indeed became significantly reduced (Figure 3I). These results suggested that a combination regimen-induced PXR-mediated degradation of Tip60 ultimately decreased acetylation of α-tubulin. The acetylation status of α-tubulin will lead to altered dynamics of tubulin and ultimately impair cellular mitosis [8]. Indeed, immunofluorescence microscopy revealed that cells treated with either SPA70 or the combination regimen displayed multipolar spindle apparatus (Yellow arrow, “T” type) and bridged α-tubulin (Figure 3J). The proportion of multipolar nuclei in the SPA70 group was 12% (*p* = 0.002), whereas the combination group increased to 17% (*p* = 0.003). Figure 3K graphically illustrates the mechanisms by which PXR interacts physically and functionally with Tip60 and regulates Tip60 activity in PTX-resistant lung cancer cells. Combination treatment with SPA70 facilitates Tip60 degradation by PXR, thus inhibiting α-tubulin acetylation. Consequently, the decreased acetylation of α-tubulin affects tubulin dynamics and induces mitosis defect. Misassembled spindles led to chromosome mis-segregation and abnormal numbers of chromosomes, which are characteristic of mitosis catastrophe. Indeed, we also observed some multinucleated giant cells, which indicated that either SPA70 or the combination regimen induces mitosis catastrophe, leading to cell death, including cell apoptosis and necroptosis, thus sensitizing PTX-resistant lung cancer cells and overcoming drug resistance.

### 3.4. Combination Treatment Produces Apoptosis and Increases S-Phase Arrest in PTX-Resistant Cells

**Figure 4:**
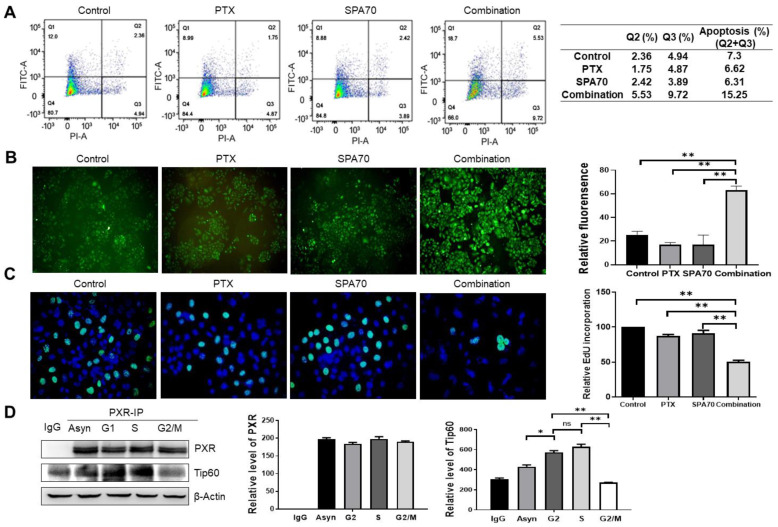
Combination treatment induces apoptosis and compromises EdU incorporation. All the experiments were conducted with 2 nM PTX, 10 μM SPA70, or combined usage for 48 h. (**A**) Combination regimen induced the highest apoptosis in A549/TR cells within all groups as shown by annexin V-FITC/PI staining. Apoptosis fraction was calculated by the summarization of coverage percentage in Q2, and Q3. (**B**) Combination regimen produced the highest ROS level in A549/TR cells among all groups by DCFH-DA probe incubation with live cells. The photographs were taken by fluorescence microscopy (200×). (**C**) Combination regimen inhibited EdU incorporation in A549/TR cells among all groups. Merged images with green (EdU-488) and blue (DAPI) were displayed and EdU fluorescence staining was quantified. The photographs were taken by fluorescence microscopy (200×). (**D**) Combination regimen-treated cells were held in different cell cycles and precipitated with the indicated antibody. Western blot detection shows increased interaction between Tip60 and PXR mainly in S-phase. *, *p* < 0.05; **, *p* < 0.01, compared with indicated groups. Ns means no statistical significance.

To further explore the mechanism of observed cytotoxic effects, next we investigated the capabilities of the indicated compounds to induce apoptosis. While cells treated with the vehicle control in A549/TR showed 7.3% apoptotic population, treatment with paclitaxel, SPA70, or PTX plus SPA70 showed 6.62, 6.31% and 15.25% apoptotic fractions, respectively (Figure 4A). Since the oxidative stress with elevated ROS induced by effective therapeutics is critical for the apoptotic ending of cancer cells, we analyzed the ROS levels in the resistant cells under different treatment conditions by staining with DCFH-DA fluorescence dye, and our results showed a dramatic increase of ROS production in the combination group (Figure 4B). In order to test whether the combination regimen directly affected the cell cycle in PTX-resistant NSCLC cells, FACS analyses were performed using PI staining. Although a similar G2/M cell cycle arrest presented in every treatment, a mildly increased S-phase arrest occurred in combination group (Supplemental Figure S3). Correspondingly, the dramatically lower Edu incorporation in the S-phase (48.6% of control group) was observed in this combination administration group (Figure 4C). In order to further investigate whether combination treatment-induced S-phase arrest was associated with PXR-Tip60 interaction, we first treated cells with PTX and SPA70 together for 24hs, then arrested cells in specific cell cycles with deoxyribonucleotide thymine (Tdr) and nocodazole, using PXR antibody to precipitate Tip60, and the result demonstrated that the interaction between PXR and Tip60 indeed increased in the S-phase (Figure 4D).

### 3.5. Combination Regimen Induces Necroptosis in Lung Cancer Cells

**Figure 5:**
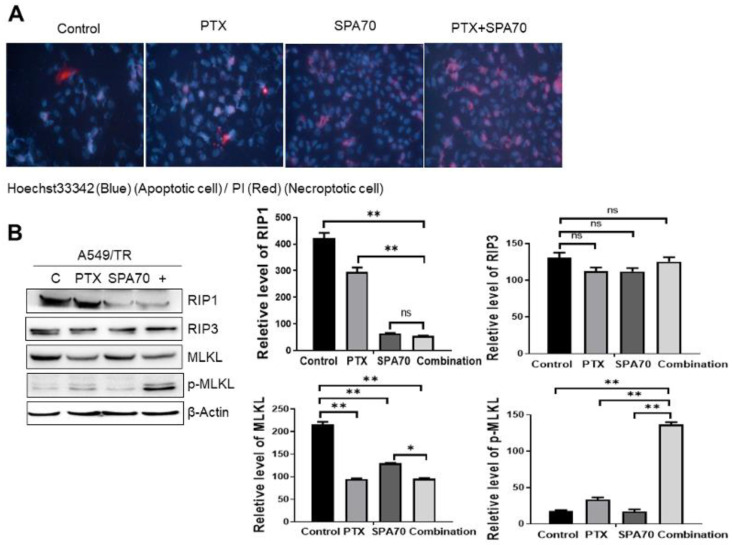
SPA70 and combination regimen induces necroptosis and exacerbates cell death. All the experiments were treated with 2 nM PTX, 10 μM SPA70, or combined regimen for 48 h. (**A**) Merge pictures showing Hoechst 33342 (specifically stains apoptotic cells) and PI (specifically stains necroptotic cells) staining in all indicated groups. The photographs were taken by fluorescence microscopy (200×). (**B**) Western blot analysis of classic RIP1-RIP3-MLKL pathway in all treatment groups (*n* = 3). “+” indicates combination treatment of PTX and SPA70. *, *p* < 0.05; **, *p* < 0.01, compared with indicated groups. Ns means no statistical significance.

It is well-documented that PTX induces G2/M arrest, thus leading to apoptosis [43]. However, we observed that SPA70 treatment seems to not induce much cell death neither in PTX-sensitive nor in PTX-resistant lung cancer cells (Figure 1B, Supplemental Figure S1A). This leads us to explore cell death mechanisms other than apoptosis. Necroptosis is a kind of programmed, regulated necrosis and it is mediated by the RIPK1 (receptor-interacting protein kinase 1, RIP1)-RIPK3 (RIP3)-MLKL (mixed lineage kinase domain-like pseudo kinase) pathway that regulates necrotic cell death [44]. TNFα was viewed as a critical proinflammatory cytokine leading to the activation of the RIPK1 kinase, subsequently promoting cell death through either necroptosis or apoptosis. Activated RIPK1 forms complex IIb with RIPK3, which subsequently activates and phosphorylates MLKL, inducing necroptosis [44]. Phosphorylated MLKL traffics from cytosol to the damaged plasma membranes, leading to necroptosis, thus MLKL serves as an obligate effector determining the process of necroptosis [45]. In order to investigate whether SPA70 could induce necroptosis in PTX-resistant lung cancer cells, we double-stained cells with Hoechst 33342 (for apoptotic cells) and PI (for necroptotic cells). We found that the combination regimen indeed exhibited strong staining of Hoechst 33342 and PI, indicating both apoptosis and necroptosis occurred in these groups (Figure 5A). Additionally, SPA70 profoundly induced necroptosis, together with significantly higher TNFα mRNA expression compared with the control group and PTX group (Supplemental Figure S4). The levels of the critical necroptosis factors were determined by Western blotting, and marked RIP1 inhibition and elevated p-MLKL were found in the combination regimen-induced cells undergoing necroptosis (Figure 5B), suggesting activation of RIP1-RIP3-MLKL necroptosis pathways.

### 3.6. Combination of PTX and SPA70 Additively Suppresses PTX-Resistant Tumor Xenograft Growth In Vivo

**Figure 6:**
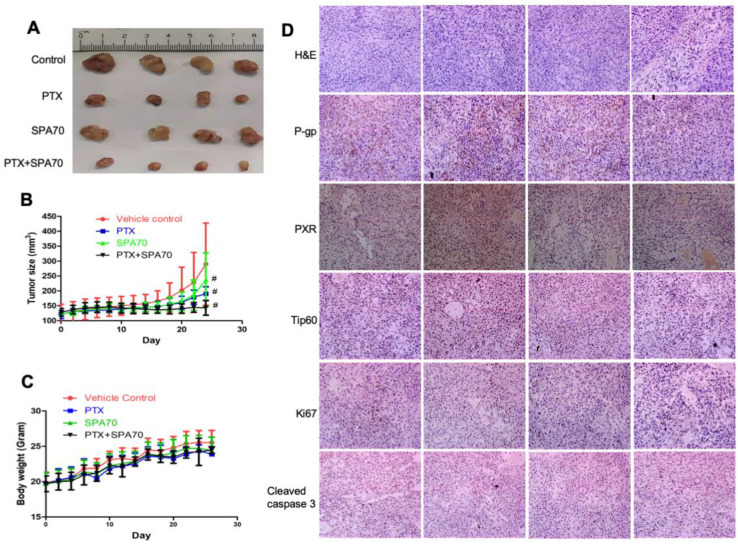
Additive effect of PTX and SPA70 in PTX-resistant A549/TR xenograft tumor growth. BALB/c-Nude mice were treated with 5 mg/kg paclitaxel with or without SPA70 intraperitoneal injection three times per week. (**A**) The photograph of xenograft tumors in all groups. (**B**) The volume of the xenograft tumors from the combination group was remarkably smaller than the vehicle control and single compound groups. #, *p* < 0.05, compared with vehicle administration groups. (**C**) Mice body weight curves over drug administration time period. (**D**) H&E and IHC staining of tumor tissues revealed remarkably reduced expressions of P-gp, along with diminished PXR and Tip60 expression, decreased proliferation (Ki67), and increased cell apoptosis (cleaved caspase 3) in the combination treatment group. The photographs were taken by microscopy (200×).

We further investigated the combination regimen treatment for its therapeutic effects in a mouse xenograft model. PTX-resistant A549/TR xenografts were established in BALB/c-Nude mice and then the mice received 5 mg/kg paclitaxel with or without 30 mg/kg SPA70 intraperitoneal injection for three days per week. Notably, the combination regimen dramatically repressed xenograft tumor growth compared with the other groups (*p* < 0.05) (Figure 6A,B). During administration, the body weight of mice in all groups did not show significant alteration (Figure 6C). PTX treatment showed slightly better tumor growth inhibition (TGI), about 58.4% more than SPA70 single treatment (TGI 32.0%), whereas the combination treatment significantly repressed tumor growth by 89.5% after 4 weeks of continuous administration to the PTX-resistant xenograft tumor model (Table 3). Of note, although the combination regimen of PTX and SPA70 showed a synergistic effect in cellular experiments, in the in vivo animal model, the combination regimen seemed to work in an additive mode, since the TGI value in the combination group (89.5%) equaled the summarization of that in both the PTX (58.4%) and SPA70 (32.0%) treatment groups. Hematoxylin and eosin (H&E) staining of tumor tissues suggested that the tumor cells had pathological alterations, including cell shape changes, together with nuclei shrinkage, some of which even lost membranes, underlining the antitumor effect of the combination regimen (Figure 6D). Immunohistochemistry (IHC) staining results suggested that tumor cell proliferation was inhibited (Ki67), cell apoptosis (cleaved caspase 3) increased, and P-gp, PXR, and Tip60 expression remarkably reduced in the combination treatment group. Overall, the above evidence proved that a combined regimen of the PXR antagonist SPA70 with PTX had a stronger inhibitory effect on PTX-resistant tumor growth than single compound treatment, indicating SPA70 might be a potent candidate to overcome PTX resistance in NSCLC cells.

### 3.7. Two SPA70 Derivatives Also Have Synergistic Effects with PTX In Vitro

**Figure 7:**
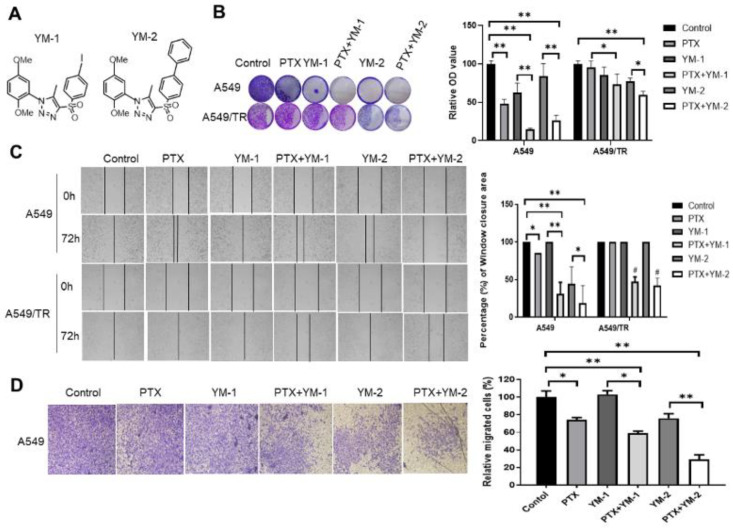
Two SPA70 derivatives also have synergistic effects with PTX in parental A549 and PTX-resistant A549/TR cells. (**A**) Chemical structure of two SPA70 derivatives YM-1, YM-2. (**B**) Representative pictures of the colonies in different groups, and the quantification of A549, A549/TR cells was shown as a bar graph (*n* = 3). A total of 1 nM PTX, and/or 5 μM YM-1/YM-2, were applied in 12-well plates with triplicates for 8–10 days. Crystal violet-stained colonies were lysed in buffer and the absorbance was recorded at 490 nm. *, *p* < 0.05; **, *p* < 0.01, compared with indicated treatment groups. (**C**) In the wound healing assay, 2 nM PTX and/or 10 μM YM-1/YM-2 were applied to the cells for 72 h. The migrated cells across the scratch line were recorded and quantified as shown in the right panel. *, *p* < 0.05; **, *p* < 0.01, compared with indicated treatment groups. The photographs were taken by microscopy (200×). (**D**) Transwell invasion assay was carried out to evaluate the inhibitory effects of YM-1 or YM-2 combined with PTX on cell invasion in parental A549 cells. The invaded cells were counted as Figure 2D. The photographs were taken by microscopy (200×). *, *p* < 0.05; **, *p* < 0.01, compared with indicated treatment groups.

As we showed above, the PXR antagonist SPA70 exhibited great potential in overcoming PTX resistance. In order to test whether the SPA70 analogues also has clinical potential, we synthesized compounds YM-1 and YM-2 based on SPA70 scaffold (Figure 7A). We combined YM-1 and YM-2 with PTX to treat parental A549 and PTX-resistant A549/TR cells. The results showed that the combination regimen synergistically suppressed colony formation of A549 and H460 (*p* < 0.05). The magnitude of suppression was higher in PTX-sensitive cell lines than those in PTX-resistant cell lines (Figure 7B). Moreover, it seems that YM-2 has a better effect than YM-1 on the inhibition of A549 cell migration, as wound healing assays demonstrated that PTX with YM-2 had 18% coverage area of control whereas treatment with PTX and YM-1 showed 32% coverage area of control (Figure 7C). In case of resistant cell lines, both YM-1 and YM-2 treatments gained comparable inhibition of migration when combined with PTX (Figure 7C). Similarly, both SPA70 analogues had inhibitory effects on cell invasion, in which YM-2 showed much potent synergistic effects with PTX (Figure 7D). Collectively, these results suggest that all of these three PXR antagonists have high potential to sensitize PTX treatment.

## 4. Discussion

Xenobiotic metabolism pathways, regulated by xenobiotic sensors such as PXR, play an important role in preventing chemical-induced damage/mutation to the genome [46,47]. Meanwhile, elevated PXR pathways also contribute to the development of therapeutic resistance. Resistance to PTX is frequently observed in cancer patients who go through long-term administration and finally fail the clinical cancer therapy. Until now, many strategies have been applied to reverse PTX resistance, however, overcoming drug resistance is still a huge challenge in a clinical setting. The mechanisms of PTX resistance in NSCLC are quite complicated, which has been thoroughly reviewed in references [8,48].

In recent years, the interactions between PXR and other proteins have attracted substantial attention, especially the interactions between PXR and histone acetyltransferase, kinase and ubiquitination enzymes. In the present study, we investigated the interactions between PXR and the HAT enzyme Tip60. Indeed, PXR binds to Tip60 at the catalytic MYST domain, overlapping with the acetyl-CoA binding site. PXR-Tip60 interaction directly led to the repression of the Tip60 HAT activity, as acetylation of α-tubulin decreased (Figure 3). This effect is similar to that of activating transcription factor 2 (ATF2), which also binds Tip60 at MYST domain spanning the acetyl-CoA binding site, thus repressing Tip60 HAT activity [49]. Indeed, posttranslational modifications (e.g., deubiquitination) of Tip60 alter its HAT activity, as ATF3-recruited USP7 was shown to stabilize Tip60 and augment its HAT activity, in which ATF3 bound Tip60 in the region adjacent of the HAT domain [42].

In the present study, PXR-inhibited Tip60 HAT activity resulted from Tip60 ubiquitination and degradation. This effect might be mediated by an unknown mechanism that PXR recruits certain E3 ligase to degrade TIP60, which is reasonable, as a study reported that the association between PXR and E3 ligase carboxy terminus of Hsc70 interacting protein (CHIP) occurred both in nuclear and the cytoplasm [50]. Phosphorylated PXR further recruited CHIP to regulate cancer cell autophagy [50]. Alternatively, PXR may bear an undefined function that affects Tip60 stabilization. Structurally, the DNA binding domain of PXR is composed of two zinc finger RING sequences, which is quite similar to that in the peroxisome proliferators-activated receptors γ (PPARγ), and PPARγ was proved as an E3 ligase to degrade NF-κB through RING region [51]. Our recent study demonstrated that PXR negatively regulates E3 ligase MDM2 protein expression [20]. However, whether the zinc finger RING sequences can endow E3 ligase activity to PXR remains to be investigated. In line with these findings, the negative regulation of Tip60 by PXR was visualized in a PTX-resistant NSCLC model, and the interaction existed in all phases of the cell cycle, especially the S-phase, suggesting that PXR-regulated Tip60 protein post-translational modifications contribute to the development of PTX resistance, and might be the potential target to overcome PTX-resistance. Interestingly, Tip60 as a tumor suppressor promotes DNA damage response and regulates homology recombination (HR) to promote DNA repair [42,52].

The direct outcome of the inhibition of HAT activity of Tip60 is the decrease of the acetylation of α-tubulin. The status of α-tubulin acetylation determined the dynamics of tubulin during mitosis. In the case of the combined application of PTX and a PXR antagonist, SPA70, microtubule dynamics were dampened, and multipolar spindle apparatus appeared thus to inhibit normal chromosomal segregation. Our current experimental results emphasized that the alteration of α-tubulin acetylation is very important in the sensitization of PTX-resistant cancer cells. Most importantly, we speculated SPA70 to be an antagonist of PXR bearing post-translational modification capability on Tip60, as indicated by increased interaction between PXR and Tip60, and decreased Tip60-acetylated α-tubulin (Figure 3).

PXR is well known for its promiscuous interaction with the broad spectrum of structurally diverse ligands, and as such it is an important factor contributing to drug resistance. Targeting PXR, therefore, is an important route for overcoming drug resistance [25]. Antagonists of PXR show great potential to overcome drug resistance. For example, PTX combined with resveratrol can sensitize PTX-resistant breast cancer cells, and sulforaphane combined with PTX can overcome PTX resistance [53,54]. However, the toxic effects of PXR antagonists cannot be avoided [55,56]. For instance, ketoconazole can inhibit the expression of PXR and the hepatoxicity of ketoconazole attracted much attention [57]. In this experiment, given that SPA70 has low toxicity [23], we studied the synergistic effects of PTX and SPA70 on PTX-resistant NSCLC cells in vitro and in vivo. Our experiment demonstrated that combination treatment with PTX and SPA70 has multifaceted mechanisms to sensitize PTX-resistant lung cancer cells. When PTX and SPA70 are combined, SPA70 disassociates PXR from the *MDR1* promoter, abrogating PXR-induced *MDR1* expression, thus overcoming drug resistance (Figure 2).

In line with the observance of mitosis defect, apoptosis and RIP1-RIP3-MLKL-mediated necroptosis was implicated in the mechanism of combination regimen-induced cell death. We found that SPA70 not only induced S-phase genetic toxicity but also produced a remarkable ROS level (Figure 4). Given that SPA70 displays promising potential in overcoming PTX resistance and it has been granted a patent, we started with sulfonyl chloride SI-1 and synthesized two analogues of SPA70. These derivatives are also PXR antagonists and exhibited a synergistic effect with PTX in both parental and PTX-resistant lung cancer cell lines. These results strongly indicated that SPA70 and its derivatives as PXR antagonists have promising potential in overcoming drug resistance.

Future research directions will focus on more specific PXR reversal agents bearing capabilities of posttranslational modifications on biomarker proteins in multi-drug resistance, thus regulating cell cycles and sensitizing the resistant cells to death. Nowadays, rational drug combination strategies have displayed substantial therapeutic effects, in particular in malignant tumors, and it is hoped that these novel findings bear huge potential to overcome clinical drug resistance and finally benefit patients.

## Figures and Tables

**Table 1:** IC_50_ of PTX and SPA70 against human NSCLC cell lines (mean ± SEM, *n* = 4).

	PTX IC_50_ (μM)	SPA70 IC_50_ (μM)
A549	0.01 ± 0.00	2.41 ± 0.13
A549/TR	0.50 ± 0.22	5.62 ± 0.53
H460	0.01 ± 0.00	4.63 ± 3.99
H460/TR	0.80 ± 0.20	7.10 ± 2.63
RI (A549/TR/A549)	100.4	2.33
RI (H460/TR/H460)	80	1.53

**Table 2:** Combination of PTX with SPA70 showed a synergistic effect in PTX-resistant cancer cell lines.

	A549	H460	A549/TR			H460/TR		
	CI ED_50_	CI ED_50_	CI ED_50_	CI ED_75_	CI ED_90_	CI ED_50_	CI ED_75_	CI ED_90_
Combination (PTX + SPA70)	0.91	0.12	0.03	0.01	0.004	0.99	0.05	0.002

Note: CompuSyn software was used to calculate combination index (CI). CI < 0.9 indicates synergism; 0.9 < CI < 1.1 indicates additive effect; CI > 1.1 indicates antagonism between the two drugs tested.

**Table 3:** TGI value of combination of PTX (5 mg/kg) and SPA70 (30 mg/kg) in the PTX-resistant A549/TR xenograft tumor model.

Treatment Group	TGI (100%)
Vehicle control	-
PTX	58.4 ± 9.0 ^#^
SPA70	32.0 ± 7.8 ^#^
PTX + SPA70	89.5 ± 7.2 ^#,^*

Note: ^#^, *p* < 0.05, compared with vehicle control; *, *p* < 0.05, compared with either PTX or SPA70 treatment group.

## Data Availability

Not applicable.

## Supplementary Materials

The following supporting information can be downloaded at: https://www.mdpi.com/article/10.3390/cells11193094/s1, Figure S1: The cell colony formation and migration, invasion effects of PTX, SPA70 and combination on H460, H460/TR cells. Figure S2: PXR protein expression negatively correlated with Tip60 protein expression in H460 and H460/TR cells. Figure S3: Flow cytometry (FACS) analysis using PI staining showed combination treatment induced S phase arrest in A549/TR cells after adding 2 nM PTX, 10 μM SPA70, or combined regimen for 48 h. Figure S4: SPA70 and combination regimen induced necroptosis and exacerbated cell death.
