# Supplementary material for: Combination of Paclitaxel and PXR Antagonist SPA70 Reverses Paclitaxel-Resistant Non-Small Cell Lung Cancer
Source: Cells. 2022 Oct 1;11(19):3094. doi: 10.3390/cells11193094 (PMC9563422; doi:10.3390/cells11193094)
Supplement: Supplementary file 1 [file cells-11-03094-s001.zip › cells-1849076-supplementary.pdf]

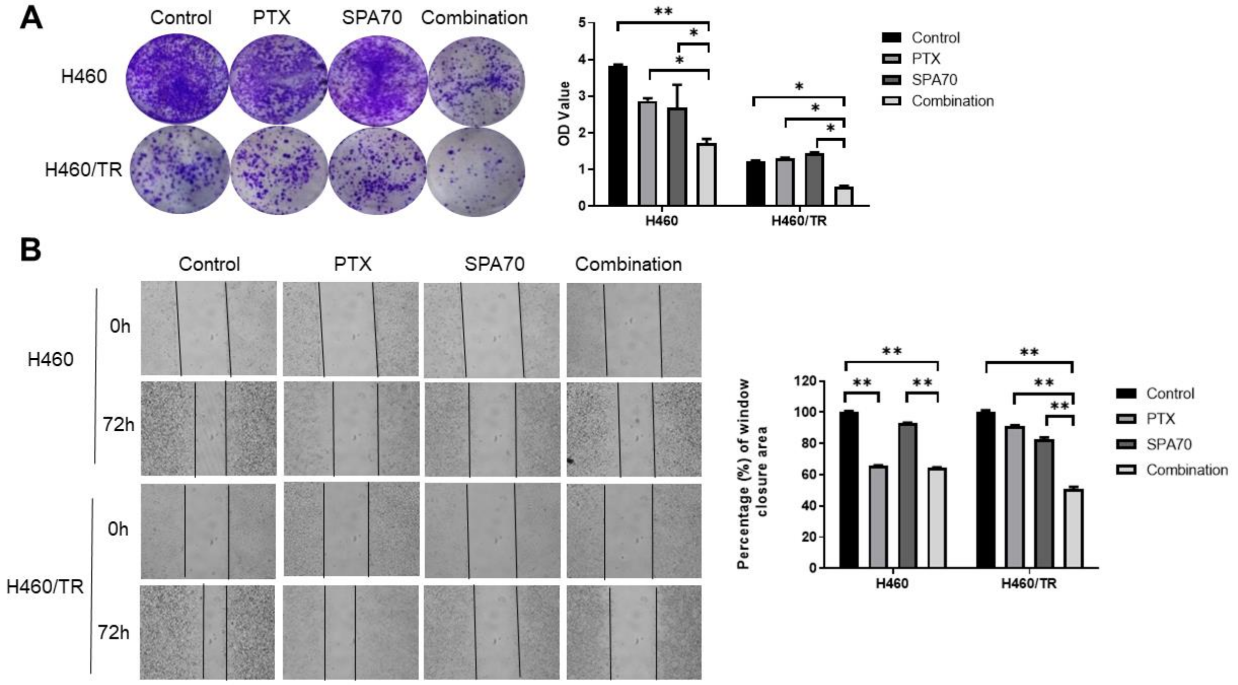

Supplemental Figure S1. The cell colony formation and migration, invasion effects of PTX, SPA70 and combination on H460, H460/TR cells. Experiment methods and statistical analysis as described in Figure 1. *, $p < 0.05$; **, $p < 0.01$, compared with indicated groups. The photographs were taken by microscopy (200 ×).

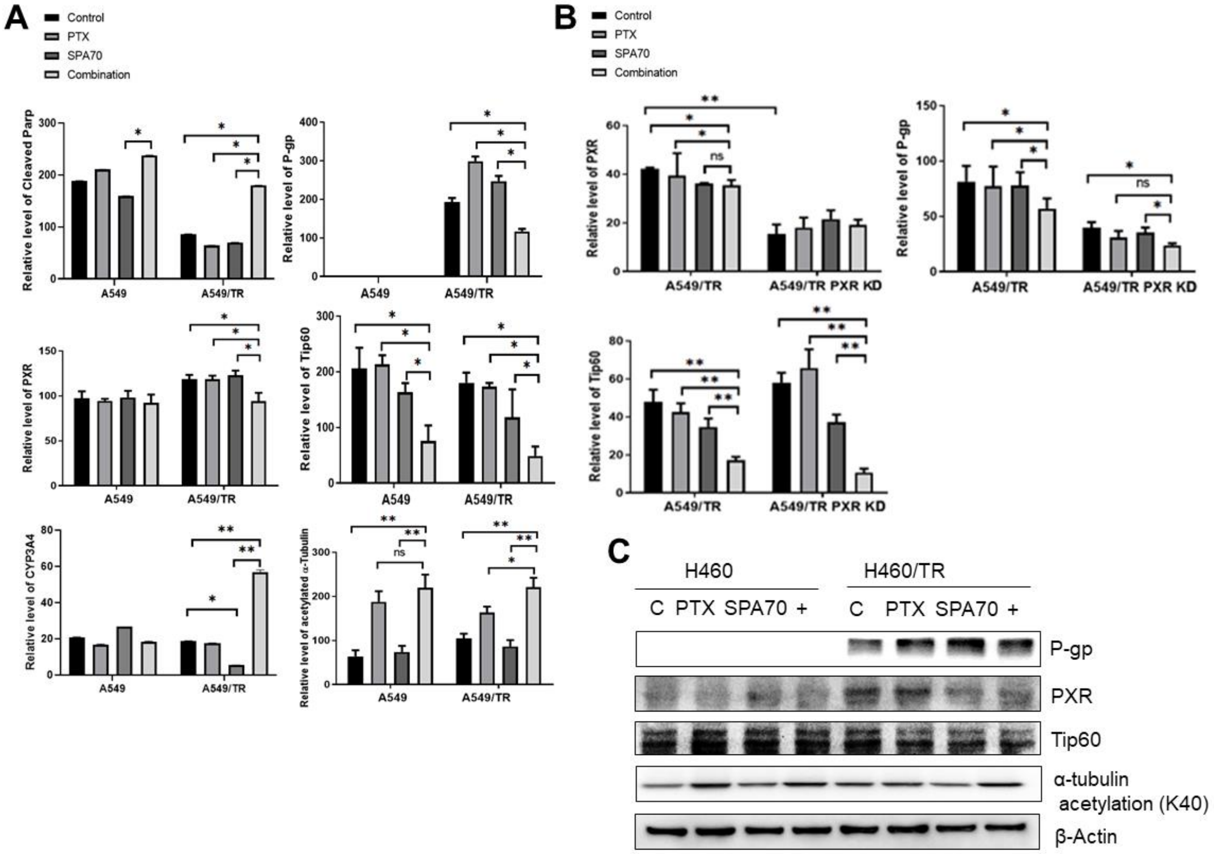

Supplemental Figure S2. PXR protein expression negatively correlated with Tip60 protein expression in H460 and H460/TR cells. **A**, Quantification of indicated treatment signaling pathway in parental A549 and PTX-resistant A549 cells in Figure 3. *, $p < 0.05$; **, $p < 0.01$, compared with indicated groups. **B**, The cells were treated with 2 nM PTX, 10 μM SPA70, or combination for 48 h (n = 3).

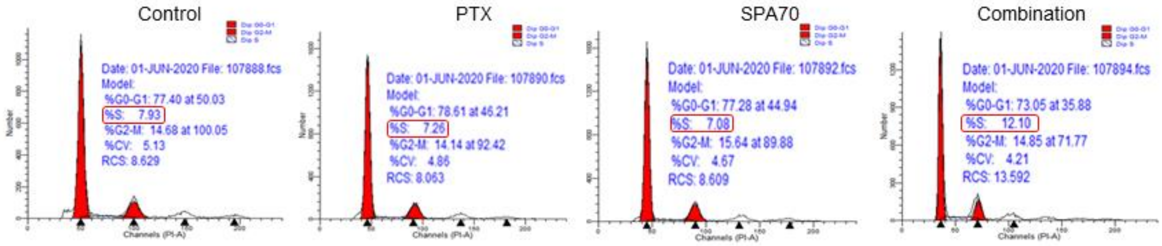

Supplemental Figure S3. Flow cytometry (FACS) analysis using PI staining showed combination treatment induced S phase arrest in A549/TR cells after adding 2 nM PTX, 10 μM SPA70, or combined regimen for 48 h.

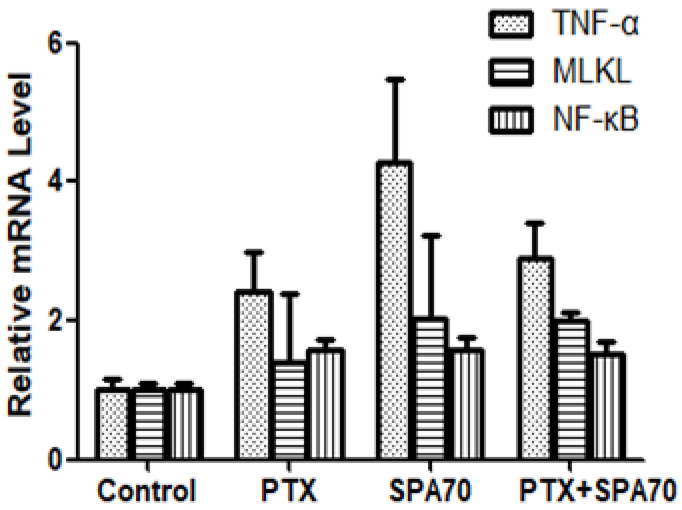

Supplemental Figure S4. SPA70 and combination regimen induced necroptosis and exacerbated cell death. Cells were treated with 2 nM PTX, 10 μM SPA70, or combined regimen for 48 h. RT-PCR was applied to determine the mRNA level of TNF-*α*, MLKL and NF-κB expression on all treatment groups.
